# Performance insights into spray-dryer microencapsulated *Bacillus thuringiensis* cry pesticidal proteins with gum arabic and maltodextrin for effective pest control

**DOI:** 10.1007/s00253-023-12990-7

**Published:** 2024-01-29

**Authors:** Jhones Luiz de Oliveira, Isabel Gómez, Jorge Sánchez, Mario Soberón, Ricardo Antonio Polanczyk, Alejandra Bravo

**Affiliations:** 1 Department of Agricultural Production Sciences, Faculty of Agronomy and Veterinary Sciences, São Paulo State University (UNESP), Jaboticabal, São Paulo, 14884-900 Brazil; 2 Present Address: Department of Molecular Microbiology, Instituto de Biotecnología, Universidad Nacional Autónoma de Mexico, Cuernavaca, Morelos Mexico

**Keywords:** *Bacillus thuringiensis*, Microencapsulation, Mechanism of action, *Spodoptera frugiperda*, *Manduca sexta*

## Abstract

**Abstract:**

*Bacillus thuringiensis* (Bt) produces crystals composed mainly of Cry pesticidal proteins with insecticidal activity against pests but are highly susceptible to degradation by abiotic factors. In this sense, encapsulation techniques are designed to improve their performance and lifetime. However, the effects of polymeric matrix encapsulation such as gum arabic and maltodextrin by spray-dryer in the mechanisms of action of Bt *kurstaki* and Bt *aizawai* are unknown. We analyzed crystal solubilization, protoxin activation, and receptor binding after microencapsulation and compared them with commercial non-encapsulated products. Microencapsulation did not alter protein crystal solubilization, providing 130 kDa (Cry1 protoxin) and 70 kDa (Cry2 protoxin). Activation with trypsin, chymotrypsin, and larval midgut juice was analyzed, showing that this step is highly efficient, and the protoxins were cleaved producing similar ~ 55 to 65 kDa activated proteins for both formulations. Binding assays with brush border membrane vesicles of *Manduca sexta* and *Spodoptera frugiperda* larvae provided a similar binding for both formulations. LC_50_ bioassays showed no significant differences between treatments but the microencapsulated treatment provided higher mortality against *S. frugiperda* when subjected to UV radiation. Microencapsulation did not affect the mechanism of action of Cry pesticidal proteins while enhancing protection against UV radiation. These data will contribute to the development of more efficient Bt biopesticide formulations.

**Key points:**

*• Microencapsulation did not affect the mechanisms of action of Cry pesticidal proteins produced by Bt.*

*• Microencapsulation provided protection against UV radiation for Bt-based biopesticides.*

*• The study’s findings can contribute to the development of more efficient Bt biopesticide formulations.*

**Graphical Abstract:**

**Figure:**
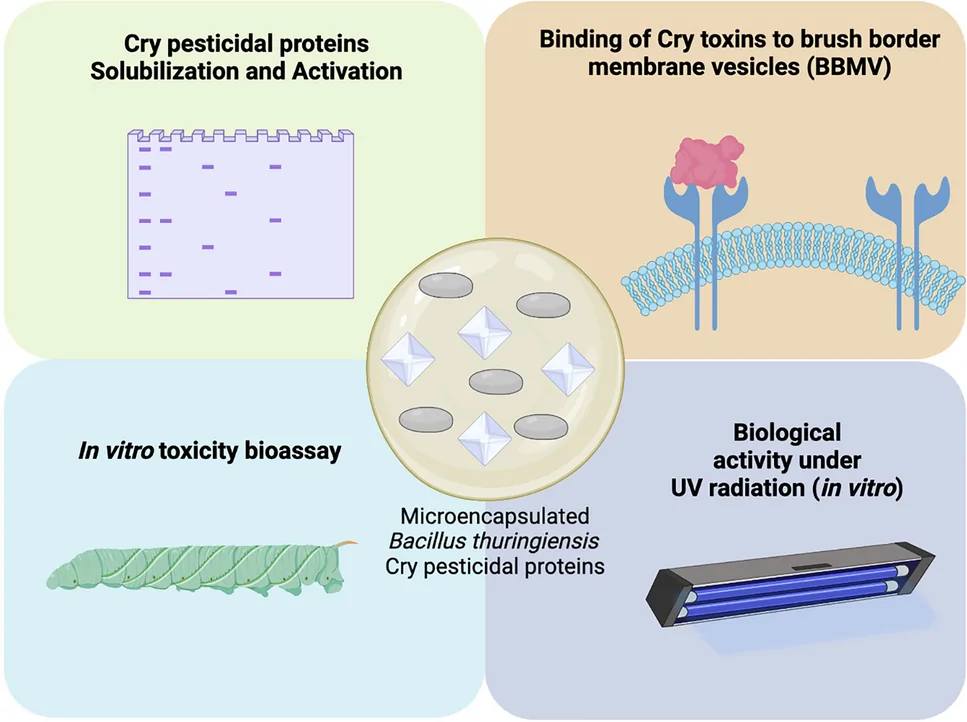

**Supplementary Information:**

The online version contains supplementary material available at 10.1007/s00253-023-12990-7.

## Introduction

Agricultural production is fundamental to the economy of many countries. The expanding human population associated with environmental changes has risen the pressure to increase agricultural food production to satisfy its growing demand (Bruinsma 2017). The green revolution was based mainly on the use of synthetic pesticides and fertilizers, which led to significant changes in the agricultural sector. However, over time, many insect pests have developed resistance to the chemicals used for their control (Tabashnik et al. 2014; Brevik et al. 2018; Richardson et al. 2020). Moreover, the excessive use of these products has caused harmful effects on the environment, leading to serious soil, surface water, and groundwater contamination. Currently, it is highly accepted that crop protection from insect damage using ecologically safe strategies is urgently needed (Shiva 2016; Nicolopoulou-Stamati et al. 2016; Fernandes et al. 2020). Research on biological control agents has increased in recent decades, with new products reaching the market, including microorganism-based pesticides (Lacey et al. 2015; Wakefield 2018). Among these microorganism-based products, the entomopathogenic bacterium *Bacillus thuringiensis* (Bt) has shown excellent results in insect control (Chattopadhyay et al. 2017). Bt is a gram-positive, spore-producing, rod-shaped bacterium that has been isolated from multiple ecosystems, including water, soil, insects, dust, and tree leaves (Paulino-Lima et al. 2013; Gutiérrez et al. 2019).

Bt produces parasporal crystal inclusions with insecticidal activity against different insect pests. The production of these crystals differentiates these bacteria from other *Bacillus* species. These crystals are essentially composed of δ-endotoxin proteins, which were initially called Cry, Cyt, and Vip and are responsible for the primary virulence effects of Bt pathogens (Knowles 1994; Adang et al. 2014). These Bt proteins are active against different insect orders, including Lepidoptera, Coleoptera, Hymenoptera, and Diptera, and also against nematodes (Frankenhuyzen 2009). Different families of proteins are produced by Bt bacteria, and a new classification of these proteins was recently updated (Crickmore et al. 2021). Thus, from now on, the name Cry will be used only for Bt proteins composed of three domains and classified according to their primary sequence and structure (Crickmore et al. 2021).

Microbial control agents such as Bt biopesticides are susceptible to degradation when applied to plants in the field. Most negative effects are due to exposure to adverse temperatures and continuous UV sunlight, which reduces the effectiveness of these pesticidal proteins (Fernández-Chapa et al. 2019). Studies on formulation technology have increased in this context, especially those that analyze encapsulation techniques. These strategies have as the main objective to improve the performance of biopesticides for insect control. Primarily, these studies have the following objectives: (i) to protect the formulation and shelf-life during storage; (ii) to ensure stability under field conditions, protecting against damage caused by sunlight and adverse temperatures; (iii) to increase residual activity after application; (iv) to increase contact with target larvae after application; and (v) to improve compatibility with other management strategies (Kala et al. 2020). However, the adverse effects of different matrices used for microencapsulation on the different steps of the mechanism of action of Bt toxins, such as solubilization, activation, and binding to their specific receptors located in the larval gut of susceptible insects, have never been analyzed.

In this study, we determined all those steps of the mechanism of action, comparing commercial and microencapsulated formulations. Overall, our results showed that microencapsulation of Bt formulations did not affect the mechanism of action of these formulations while enhancing protection against UV radiation.

## Material and methods

### Preparation of Bt microencapsulated systems

*B. thuringiensis aizawai* (Bta) and *B. thuringiensis kurstaki* (Btk) organisms were obtained after pasteurization and growth in the Luria broth medium of the commercial products Xentari^®^ and Dipel^®^ (Sumitomo Chemical Brazil, São Paulo, Brazil), respectively. Gum arabic (10%) and maltodextrin (10%) were used for the encapsulation procedure. The spray-drying conditions consisted of an inlet temperature of 90 °C, an outlet temperature of 50 °C, a drying flow of 1.8 m^3^ min^−1^, a feeding flow of 0.3 L h^−1^, a spray flow of 40 L h^−1^, and double sprayer of 1 mm.

### Solubilization and activation conditions of Cry proteins

We used 50 mg of each non-encapsulated formulated product (Xentari^®^ and Dipel^®^) and a 60/40 mixture of Dipel^®^/Xentari^®^, as well as the newly designed microencapsulated products (MP_Bta and MP_Btk) and a 60/40 mixture of MP_Btk/MP_Bta, dissolved in 10 mL of deionized water for 5 min. Spore/crystal mixtures were recovered by centrifugation (10 min at 12,000 *xg*), with the pellet being suspended in 2 mL ddH_2_0 and stored at − 70 °C. These pellets were considered the “total protein samples,” as stated below. A 10-µL sample from each formulation was mixed with 10 µL 2 × Laemmli sample loading buffer (0.125 mM Tris–HCl, pH 6.8, 4% SDS, 20% glycerol, 10% β-mercaptoethanol, and 0.01% bromophenol blue), heated for 3 min at 100 °C, and analyzed by loading on 10% SDS-PAGE. The protein concentration of the final spore/crystal mixtures suspended in ddH_2_0 was estimated using the method by Bradford (1976) (Bradford 1976) using a bovine serum albumin (BSA) standard curve as a reference. As controls, the Bta and Btk strains used for the microencapsulation preparation and the Bt *kurstaki* (HD-1 strain) were grown for 72 h at 30 °C in three different mediums: HCT, SP, and Embrapa (Lecadet et al. 1980; Monnerat et al. 2007; Soberón et al. 2007) to produce the parasporal-crystals. After bacterial sporulation, the spore/crystal mixtures were recovered by centrifugation 10 min at 12,000 *xg* and washed three times with washing solution (300 mM NaCl, 10 mM EDTA) and three times with PMSF 1 mM (final concentration). Subsequently, the spore/crystal mixture was suspended in ddH_2_0 and analyzed on SDS-PAGE, as described above.

The samples of total protein were incubated for 15 min in solubilization buffer (50 mM NaOH, pH 10.5 supplemented with 0.2% β-mercaptoethanol) at 4 °C for solubilization. Then, the samples were centrifuged (12,000 *xg* for 10 min), and the soluble (supernatant) and insoluble fractions (pellet) were separated and analyzed on 10% SDS-PAGE.

Toxin activation was performed using trypsin, chymotrypsin, and midgut juice from *S. frugiperda* larvae. The midgut juice sample containing the intrinsic proteases that are present in the insect’s gut lumen was obtained after dissection of the intestinal tissue and centrifugation at 12,000 *xg* for 10 min. Before activation, the pH of the solubilized samples was adjusted to pH 8.5 by adding ¼ of the volume of 1 M Tris–HCl at pH 8, and activation kinetics analyses were performed at different incubation times (15 min, 30 min, 1 h, and 2 h) with trypsin (1:20 w/w, enzyme/substrate), chymotrypsin (1:5, 2:1, and 5:1 w/w, enzyme/substrate), and *Sf*-midgut juice (5% v/v). All digestions were carried out at 37 °C, and the incubations were stopped by adding 1 mM PMSF (final concentration). Activated toxins were loaded onto 10% SDS-PAGE gels to verify the extent of digestion, and the protein concentration was determined by Bradford assay.

Solubilization efficiency and activation efficiency were calculated for the non-encapsulated and encapsulated formulations. The solubilization efficiency was obtained by analyzing the protein concentration of the soluble fraction divided by the total protein concentration in the sample, using the following formula: Solubilization efficiency (%) = (µg protein in the soluble fraction/µg protein in the total protein sample) × 100. The activation efficiency was obtained by analyzing the protein concentration of the activated sample divided by the protein concentration in the solubilized sample, using the following formula: activation efficiency (%) = (µg protein in the activated fraction/µg protein in the solubilized fraction) × 100.

The solubilization of the formulations in the presence of midgut juice was evaluated using the different samples incubated with midgut juice at different concentrations, as indicated in the text, for 1 h and 24 h and centrifugated for 10 min at 12,000 *xg*. The solubilized proteins were analyzed on 10% SDS-PAGE gels.

### Preparation of BBMVs

Brush-border membrane vesicles (BBMVs) were prepared using third instar *S. frugiperda* and *M. sexta* larvae, according to the method of Wolfersberger et al. (1987) (Wolfersberger et al. 1987), modified by Reuveni and Dunn (1991) (Reuveni and Dunn 1991). The midgut tissue was dissected from the larvae in storage buffer (300 mM mannitol, 20 mM 2-mercaptoethanol, 5 mM EGTA, 1 mM EDTA, 0.1 mM PMSF, 150 µg mL^−1^ pepstatin A, 100 µg mL^−1^ leupeptin, 1 µg mL^−1^ soybean trypsin inhibitor, 10 mM HEPES, and 2.4 µg mL^−1^ neomycin sulfate, pH 7.5), immediately frozen and stored at − 80 °C until use. The frozen midguts were mechanically homogenized in homogenization buffer (200 mM mannitol, 10 mM ascorbic acid, 5 mM EDTA, 0.03% w/v PMSF, 1% mM PVPP, 0.2 mM leupeptin, 2 mM DTT, 10 mM HEPES pH 7.4) for 10 s for BBMV preparation. A volume of 24 mM MgCl_2_ was added, and the mixture was incubated for 10 min at 4 °C. Following the mixture centrifugation (10 min, 6000 *xg* at 4 °C), the supernatant was also centrifuged (30 min, 30,000 *xg* at 4 °C), and the final pellet was suspended in 200 mM mannitol, 1 mM DTT, 1 mM HEPES–Tris, pH 7.4, and stored at − 80 °C until use.

APN and ALP activities in *Sf*BBMVs and *Ms*BBMVs were measured. APN activity was tested using L-leucine-*p*-nitroanilide as substrate, and ALP activity was tested using *p*-nitrophenyl phosphate as substrate (Arenas et al. 2010). Protein content was measured by the DC protein–dye method (Bio-Rad), using BSA as a standard (Pierce). The initial rate at 405 nm (Ultrospec II spectrophotometer; GE Healthcare) was used to calculate the specific enzymatic activity of both enzymes. The coefficient of absorption of *p*-nitroanilide was 9.9 × 10^−3^ mol L^−1^. One unit of specific APN activity was defined as the amount of enzyme that catalyzes the hydrolysis of 1 µmol of L-leucine-*p*-nitroanilide min^−1^ mg^−1^ of protein at 25 °C. One unit of specific ALP activity was defined as the amount of enzyme that produces 1 µmol of nitrophenol min^−1^ mg^−1^ of protein at 25 °C. Nitrophenol concentration was calculated using a standard curve of 4-nitrophenol in 0.5 mM MgCl_2_, 100 mM Tris, pH 9.5. APN and ALP-specific activities showed values of enrichment factors between 2.5- and 4.2-fold in the purified BBMV compared to their initial homogenate.

### Binding of Cry1 toxins to *S. frugiperda* and *M. sexta* BBMV

Binding assays of Cry1-activated toxins to BBMV from third-instar *S. frugiperda* (*Sf*BBMV) and *M. sexta* (*Ms*BBMV) larvae were performed as follows. In these assays, different concentrations (0.152–2.27 μM) of activated Cry proteins from different samples were incubated with 10 µg BBMV protein for 1 h at room temperature in 100 µL of binding buffer (PBS, 0.1%, BSA, 0.1% Tween 20, pH 7.6). A control of *Sf*BBMV and *Ms*BBMV without toxin incubation was included in these assays. After incubation, the unbound toxin was removed by centrifugation for 10 min at 12,850 *xg*. The pellet containing *Sf*BBMV or *Ms*BBMV and bound toxin was washed twice with 100-µL binding buffer, suspended in 10 µL of PBS, and mixed with 10 µL 2X Laemmli sample loading buffer. The samples were boiled for 3 min, loaded onto 10% SDS-PAGE gels, and electroblotted onto polyvinylidene difluoride membrane (PVDF) (Immobilion-P, Bio-Vin). The PVDF membrane was blocked with 0.5% milk powder and 0.1% Tween 20 for 1 h under stirring, and bound Cry1A toxins were revealed by western blot using anti-Cry1A polyclonal antibody (1/30,000 dilution; 1 h) as a primary antibody. The goat anti-rabbit antibody coupled to the horseradish peroxidase (HRP) enzyme (Santa Cruz Biotechnology, Dallas, TX, USA) (1/10,000 dilution; 1 h) was used as a secondary antibody, followed by luminol (Santa Cruz Biotechnology Inc.) treatment, according to the manufacturer’s instructions.

### ELISA binding assays

*Sf*BBMV and *Ms*BBMV proteins were used to coat 96-well ELISA plates (2.5 µg/well for *Sf*BBMV and 1.0 µg/well for *Ms*BBMV) (Rochester, NY, USA). The different activated protein samples at different concentrations (0.152–2.27 μM) were incubated with BBMV-coated ELISA plates. Unbound toxins were removed by washing with PBS buffer, followed by three washes with PBS supplemented with 0.1% Tween 20. Bound toxins were detected using an anti-Cry1A polyclonal antibody (1:20,000 dilution) and a secondary goat anti-rabbit antibody conjugated with HRP enzyme (1:20,000 dilution). Finally, *o*-phenylenediamine (Sigma) and H_2_O_2_ were used as substrates for peroxidase activity detection. The reaction was stopped by adding 50 µL of 5 M HCl, and OD_490_ was measured using an ELISA microplate reader (PerkinElmer, Waltham, MA, USA). Negative controls were performed in parallel, where BBMV proteins were not used to coat the ELISA plate wells. All experiments were conducted in triplicates and plotted using GraphPad Prism 9.

### Toxicity bioassays

Toxicity bioassays of non-encapsulated (Xentari^®^, Dipel^®^, and Dipel^®^/Xentari^®^) and microencapsulated products (MP_Bta, MP_Btk, and MP_Btk/Bta) were performed against neonate *S. frugiperda* and *M. sexta* larvae. We used the surface contamination method. Different concentrations of the formulations (0.5 to 20 µg of formulation/cm^2^ of artificial diet) were applied to the diet surface contained in 128-well polystyrene plates (Bio-BA-128 bioassay trays; C-D International, Inc.). A total of 72 larvae were used per formulation concentration (one larva per well). Mortality was recorded after 7 days; larvae were considered dead if they showed no apparent movement. As negative controls, the diet was surface contaminated with water. In these assays, the negative control exhibited mortality rates below 5%. The mean lethal concentration (LC_50_) was estimated by Probit analysis (Polo-PC LeOra Software), and the fiducial limits at each LC_50_ value were estimated.

### Effect of UV radiation of Bt formulations on the insecticidal activity against *S. frugiperda* larvae

The bioassay plates containing 14 µg of formulation/cm^2^ of the different formulations applied on the diet surface were subjected to UV exposure (254 nm) in a laminar flow for 15 min to evaluate the effect of UV exposure of Bt formulations on the toxicity against *S. frugiperda*. A negative control was also conducted by applying only water to the diet. After UV exposure, a neonate *S. frugiperda* larva was added to each plate well.

## Results

### Protein profile of Bt strains compared to Bt formulations

Samples containing Bta control strain grown in different sporulation media showed the expected Cry1 protoxin size of 130 kDa, while samples of Btk control strains showed the presence of two major protoxin protein bands of 130 and 70 kDa (Figure S1). These molecular masses correspond to the typical sizes of proteins belonging to the Cry1 and Cry2 classes (Crickmore et al. 1998; Lereclus et al. 2000), respectively. The non-encapsulated and microencapsulated formulations presented similar protein profiles to the corresponding Btk and Bta control strains (Figure S1). However, the non-encapsulated commercial products Xentari® and Dipel® had a higher concentration of total protein than the microencapsulated formulations MP_Bta and MP_Btk after suspension of similar water volume. Image analysis using the ImageJ program indicated that the Xentari formulation has 2.2-fold more protein than MP-Bta, and the Dipel formulation has 1.9-fold more protein than MP-Btk.

### Analysis of solubilization efficiency

**Fig. 1:**
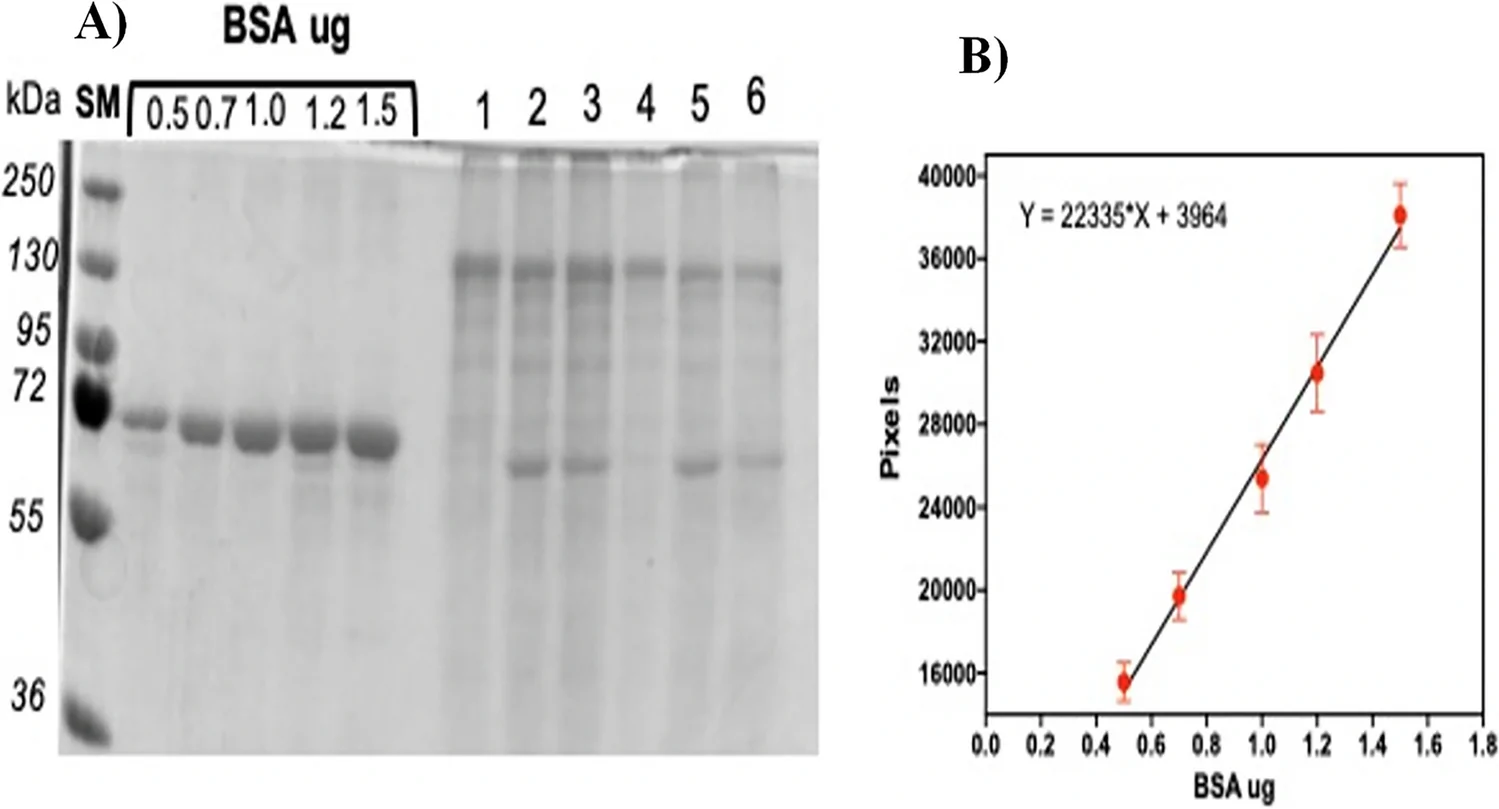
**A** Protein profile of the supernatant from formulations after solubilization in 50 mM NaOH buffer and 0.2% β-mercaptoethanol; **B** BSA control curve. Formulations: 1, Xentari^®^; 2, Dipel^®^; 3, Dipel^®^/Xentari^®^; 4, MP_Bta; 5, MP_Btk; and 6, MP_Btk/Bta

Figure 1 shows the protein profile after solubilization in 50 mM NaOH and 0.2% β-mercaptoethanol at 4 °C. Protoxin proteins of 130 and 70 kDa were solubilized under these conditions. The protein concentration in each band was estimated by using the corresponding BSA control curve, as shown in Fig. 1, and the corresponding values of solubilized proteins from the formulations are shown in Table 1.

**Table 1:** Protein quantification of formulations and solubilization efficiencies

Formulations	Formulations in water (µg)	Water supernatant discarded (µg)	Solubilized—pellet (µg)	Solubilized—supernatant (µg)	Solubilization efficiency (%)
1—Xentari^®^	.2.13	0.28 µg	0.178	0.49	26.50
2—Dipel^®^	1.33	0.15	0.069	0.66	55.93
3—Dipel®/Xentari^®^	1.43	0.13	0.014	0.62	47.69
4—MP_Bta	1.49	0.06	0.020	0.34	23.86
5—MP_Btk	1.16	0.10	0.021	0.50	47.28
6—MP_Btk/Bta	1.52	0.12	0.009	0.62	44.22

These data indicate that solubilization is not an efficient step, as solubilization efficiency values ranged from 23 to 55%. We selected to work with this extreme condition because it was more efficient for the Cry2 protein solubilization than the solubilization in 50 mM bicarbonate buffer pH 10.5 supplemented with 0.2% β-mercaptoethanol, in which the 70 kDa protein from Dipel® formulation, corresponding to Cry2Ab, was not observed as a solubilized protein (data not shown).

It shows that the solubilization efficiency of Xentari^®^ and Dipel^®^ formulations was slightly higher than those of MP_Bta (0.11-fold higher) and MP_Btk (0.18-fold higher), respectively. Furthermore, protoxin solubilization from formulations made with Btk was approximately twofold (1.92–2.11-fold) more efficient than formulations with Bta.

We also analyzed the solubilization process using midgut juice from *S. frugiperda*. For this step, a standardization of gastric juice concentration was initially performed (data not shown). Different concentrations ranging from 0.5 to 50% v/v of midgut juice were tested, with a concentration of 30% v/v being selected for the following tests. The solubilization was carried out for 1 h and 24 h. Importantly, the gastric juice was extracted from third-instar *S. frugiperda* larvae and showed a pH of 9.5. Water addition for the dilution of midgut juice did not change the pH of this solution.

**Fig. 2:**
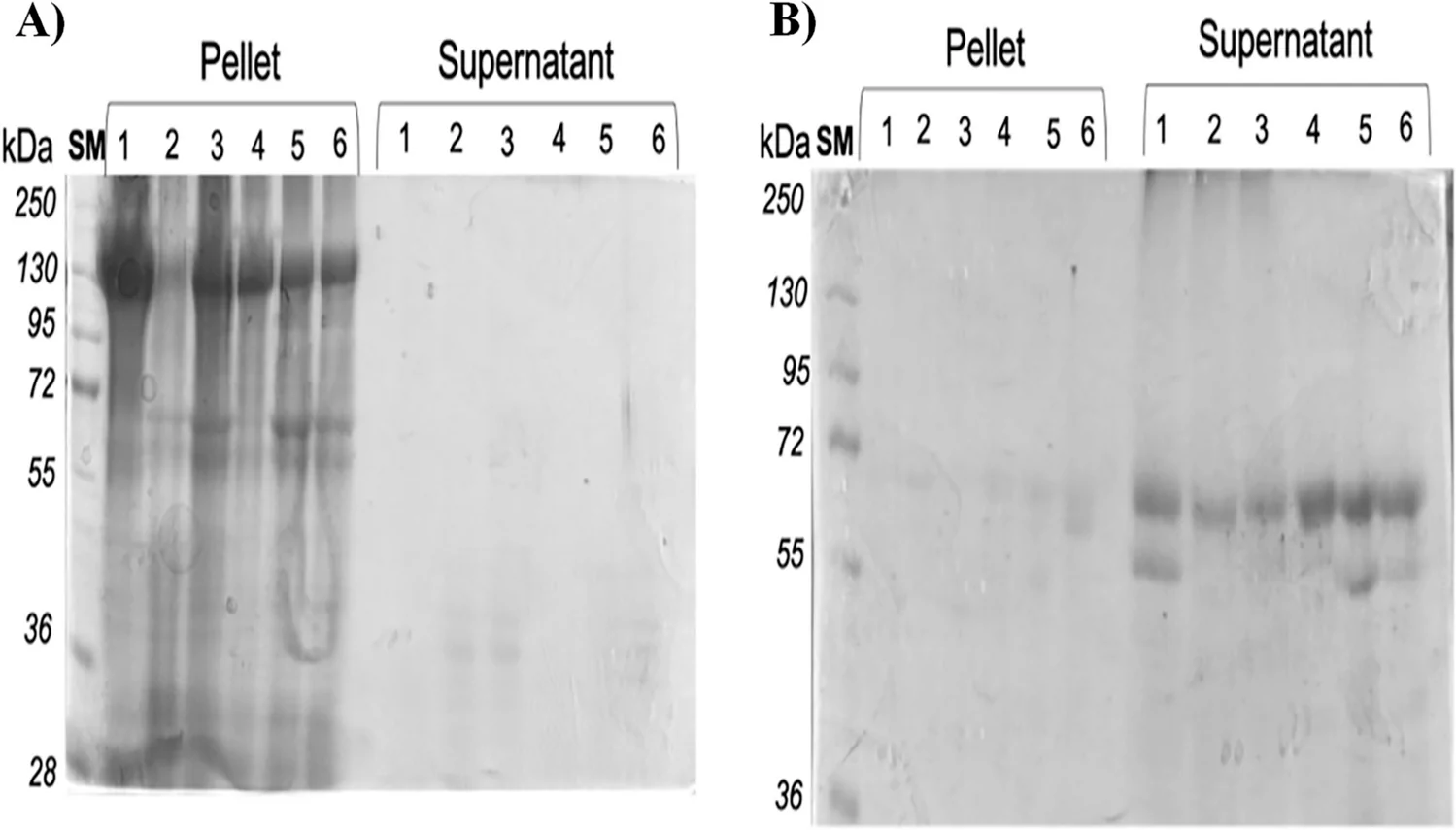
Evaluation of the protein profiles on SDS-PAGE of different formulations after solubilization in midgut juice (30% v/v) isolated from *S. frugiperda* larvae. **A** Protein profile of formulations incubated in midgut juice pH 9.5 for 24 h; **B** protein profile of formulations incubated in midgut juice pH 10.5 for 24 h. Formulations: 1, Xentari^®^; 2, Dipel^®^; 3, Dipel®/Xentari^®^; 4, MP_Bta; 5, MP_Btk; and 6, MP_Btk/Bta

Figure 2 shows the protein profile on SDS-PAGE gel after solubilization with larval midgut juice. Formulations incubated with larval midgut juice showed no protein solubilization even after 24 h, and protein bands were observed only in the pellet samples (Fig. 2A). These data indicate that solubilization may be a critical step inside the larva. Then, we made a pH adjustment of the gastric juice using 50 mM NaOH solution to reach a pH of 10.5. Under this condition, 1 h incubation was not enough to solubilize Cry proteins, as the ~ 130 kDa protein band was only observed in the pellet (data not shown). However, complete solubilization was observed when the incubation time was increased up to 24 h (Fig. 2B). We did not observe protein bands in the pellet, and protoxin proteins were found activated in the supernatant, where protein bands of ~ 55 to 60 kDa were observed for all formulations. These proteins were processed by endogenous proteases, which are present in the midgut fluid of insects. Microencapsulation did not influence the solubilization profile compared to non-encapsulated samples. However, image analysis with the ImageJ program indicated that the concentration of solubilized-activated toxin in microencapsulated samples was 0.2–0.5-fold higher than in the commercial non-encapsulated sample, indicating that solubilization was improved in microencapsulated samples.

### Analysis of activation efficiencies

The most abundant proteolytic enzymes in the midgut of insects are serine proteases, such as trypsin and chymotrypsin (Liu et al. 2010). Therefore, in vitro activation assays were performed with trypsin, chymotrypsin, and midgut juice isolated from *S. frugiperda*. Incubation time kinetics (15 min, 30 min, 1 h, and 2 h) and concentration tests varying the enzyme/protein ratios for chymotrypsin (1:5, 2:1, and 5:1) were performed. We used a ratio of 1:20 for trypsin (enzyme/protein w/w), while a concentration of 5% v/v was used for the midgut juice.

The standardization results showed that the toxin activation process for all tested enzymes occurred after 15 min of incubation, while the incubation times for the subsequent tests were only for 15 min and 1 h. The 2:1 and 5:1 ratios (enzyme/protein w/w) showed no differences in the protein profile for chymotrypsin, and the 2:1 ratio was selected for the following tests.

Figure S2 shows the SDS-PAGE gels with the protein profiles after activating the different formulations with trypsin (Figure S2-A), chymotrypsin (Figure S2-B), and midgut juice (Figure S2-C). We observed no differences in the protein profiles of the activated toxins from microencapsulated formulated products compared to those non-encapsulated (Figure S2). Table 2 shows the activation efficiencies after analyzing the intensity of bands using the ImageJ program. These data showed that the activation process is more efficient than solubilization, as up to 96% activation efficiency was observed for some samples. Moreover, both types of formulations, microencapsulated and non-encapsulated, showed rather similar values. In general, non-encapsulated products were slightly less efficiently activated than microencapsulated formulations when treated with single commercial proteases, such as trypsin and chymotrypsin. For example, when trypsin was used as protease, a 0.1-fold higher activation efficiency was observed for MP_Bta than the Xentari^®^ formulation, and MP_Btk showed a 0.18-fold higher activation efficiency than Dipel^®^. However, microencapsulated formulations showed a slightly lower activation efficiency than commercial non-encapsulated products when midgut juice was used (0.10-fold lower for MP-Bta vs Xentari^®^ and 0.18-fold lower for MP-Btk vs Dipel^®^).

**Table 2:** Protein quantification of formulations and calculation of activation efficiencies

Formulations	Solubilized (µg)	Trypsin (µg)	Chymotrypsin (µg)	Midgut juice (µg)	Trypsin activation efficiency (%)	Chymotrypsin activation efficiency (%)	Midgut juice activation efficiency (%)
1—Xentari^®^	0.49	0.42.2 g	0.28	0.35	84.83	58.16	72.20
2—Dipel^®^	0.66	0.54	0.40	0.44	81.86	60.30	66.11
3—Dipel®/Xentari^®^	0.62	0.53	0.32	0.38	86.23	51.93	62.08
4—MP_Bta	0.34	0.32	0.19	0.24	93.80	55.59	66.74
5—MP_Btk	0.50	0.48	0.29	0.28	96.73	58.00	55.98
6—MP_Btk/Bta	0.62	0.48	0.30	0.33	88.18	47.74	53.07

### Binding of toxins from conventional and microencapsulated formulations to BBMV from *S. frugiperda* and *M. sexta* biological activity of the different formulations was

**Fig. 3:**
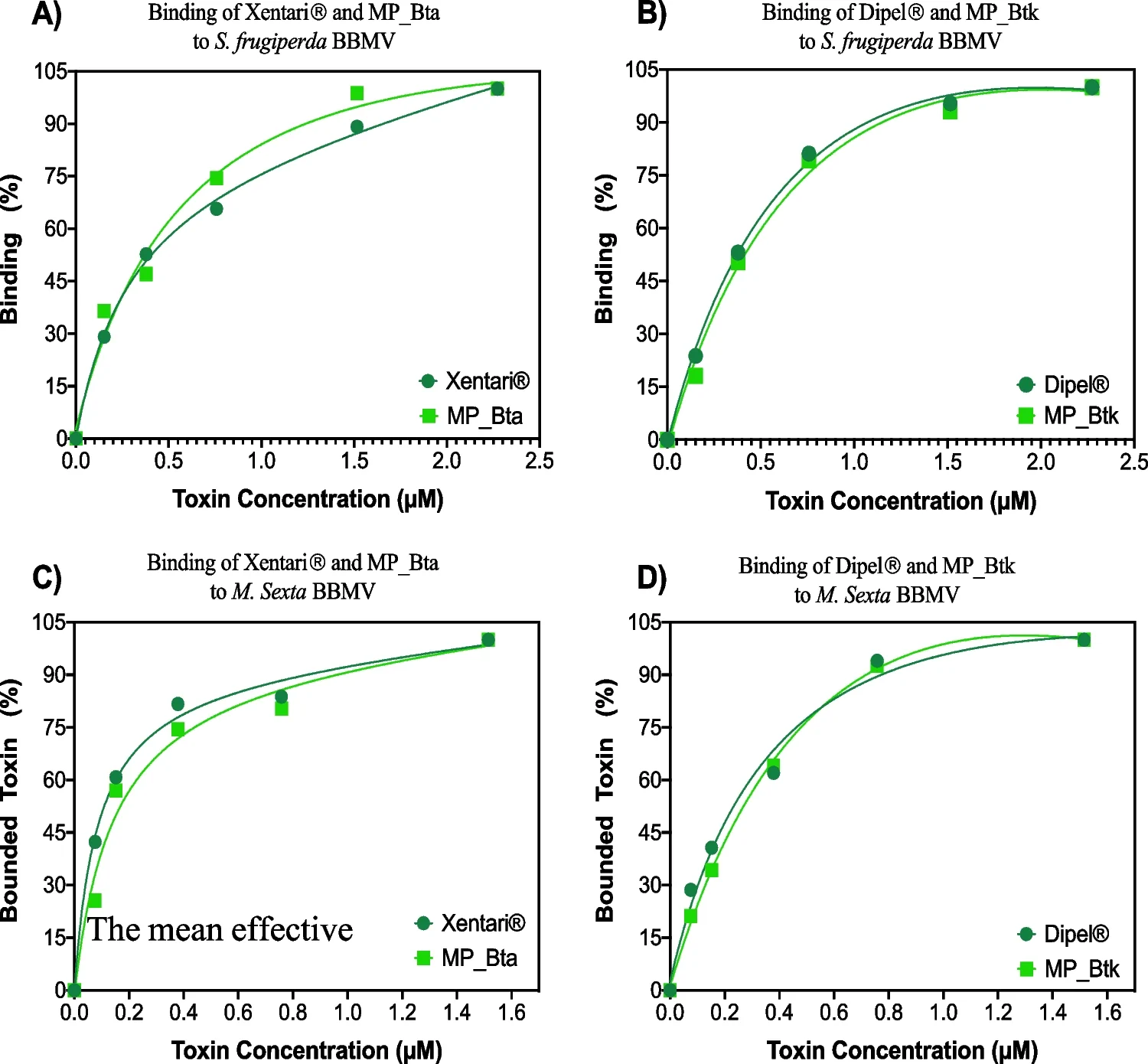
Binding interaction of activated toxin samples from different formulations to BBMV from *S. frugiperda* (**A**, **B**) and *M. sexta* (**C**, **D**). A sample of 10 µg BBMV was incubated with different concentrations of activated toxins present in different formulations. Plots were constructed after densitometry analysis of the 65 kDa band shown in Figure S3

Figure S3 shows the results of binding the Xentari^®^ and Dipel^®^ formulations and their respective microencapsulated formulations (MP_Bta and MP_Btk) with BBMVs isolated from *S. frugiperda* and *M. sexta* midgut tissue samples. For these assays, the activated samples of the formulations were incubated with BBMV from the different insects. After incubation, the BBMV-bound toxin was recovered by centrifugation, washed, and analyzed in the western-blot analysis. The binding of activated toxins from different formulations to *Sf*BBMV (Figure S3-A and B) and *Ms*BBMV (Figure S3-C and D) is directly correlated with the concentration of the used toxin. Figure 3 shows the binding analysis after the densitometry of bands by the ImageJ program. Receptor binding was calculated by normalizing to the intensity of the band, corresponding to the formulation alone, without the presence of BBMV (data not shown).

The mean effective concentration (EC_50_) value in these binding experiments is the concentration of ligand at which half of the target is present in the bound state (Table 3).

**Table 3:** EC_50_ values for conventional and microencapsulated formulations evaluated with *M. sexta* and *S. frugiperda* BBMV

	EC_50_ values (μM)	
	*Manduca Sexta*	*Spodoptera frugiperda*
Xentari^®^	0.08	0.29
Dipel^®^	0.32	0.94
MP_Bta	0.14	0.67
MP_Btk	0.75	1.12

The lower EC_50_ values found for the assays with *M. sexta* BBMV indicate a higher binding affinity than for *S. frugiperda* BBMV, which corroborates previous data (Gómez et al. 2018), which indicates a higher susceptibility of *M. sexta* to Cry1A toxins compared to *S. frugiperda*.

These results also show that Cry1A proteins from conventional formulations have higher affinity than microencapsulated formulations, as they showed lower EC_50_ values. However, the activated toxin samples used in these assays contain a mixture of proteins and, therefore, affinity values consist of apparent values that cannot be adjudicated to a single protein. In addition, Fig. 3 shows that the binding curves for both types of formulations were highly similar, proving that there were no significant variations in the binding process to BBMV.

ELISA binding assays were also performed between the different formulations and *Sf*BBMV and *Ms*BBMV to confirm these results (Figure S4). The data show similar binding curves, confirming that samples from microencapsulated products have similar binding to BBMV than samples from non-encapsulated formulations.

### Toxicity bioassays

The biological activity of the different formulations was assayed against *S. frugiperda* and *M. sexta* larvae. Table 4 shows the mean lethal concentration (LC_50_) values. The results of the controls (application of only water to the diet) showed mean mortality rate of 3 ± 0.6%.

**Table 4:** Insecticidal activity of different biological formulations (conventional and microencapsulated) against first-instar *S. frugiperda* and *M. sexta* larvae

Formulations	LC_50_ values in μg/cm^2^ (fiducial limits)^a^	
Biological	*Spodoptera frugiperda*	*Manduca sexta*
Xentari^®^	0.72 (0.49–1.20)	0.74 (0.54–1.04)
Dipel^®^	1.11 (0.68–1.82)	0.68 (0.51–0.95)
Dipel^®^/Xentari^®^	1.01 (0.68–1.47)	1.09 (0.82–1.56)
MP_Bta	0.61 (0.44–0.85)	0.84 (0.61–1.20)
MP_Btk	1.16 (0.82–1.64)	0.47 (0.33–0.63)
MP_Btk/Bta	1.06 (0.69–1.62)	0.80 (0.58–1.14)

^a^Means of at least three repetitions; fiducial limits consist of 95% confidence intervals

The results shown in Table 4 indicate no significant differences between commercial products and microencapsulated products for biological formulations. In contrast, MP-Btk for *M. sexta* showed higher potency than MP-Bta, while the opposite was found for *S. frugiperda* larvae, as MP-Bta showed higher potency than MP-Btk. These tests were carried out with a diet and the same condition was applied to all treatments, without exposure to external factors. However, formulated products may improve effectiveness under environmental conditions, such as UV radiation and high temperature, among others. In this context, we decided to carry out tests by applying an external factor such as UV light to evaluate the effect of these formulations.

**Fig. 4:**
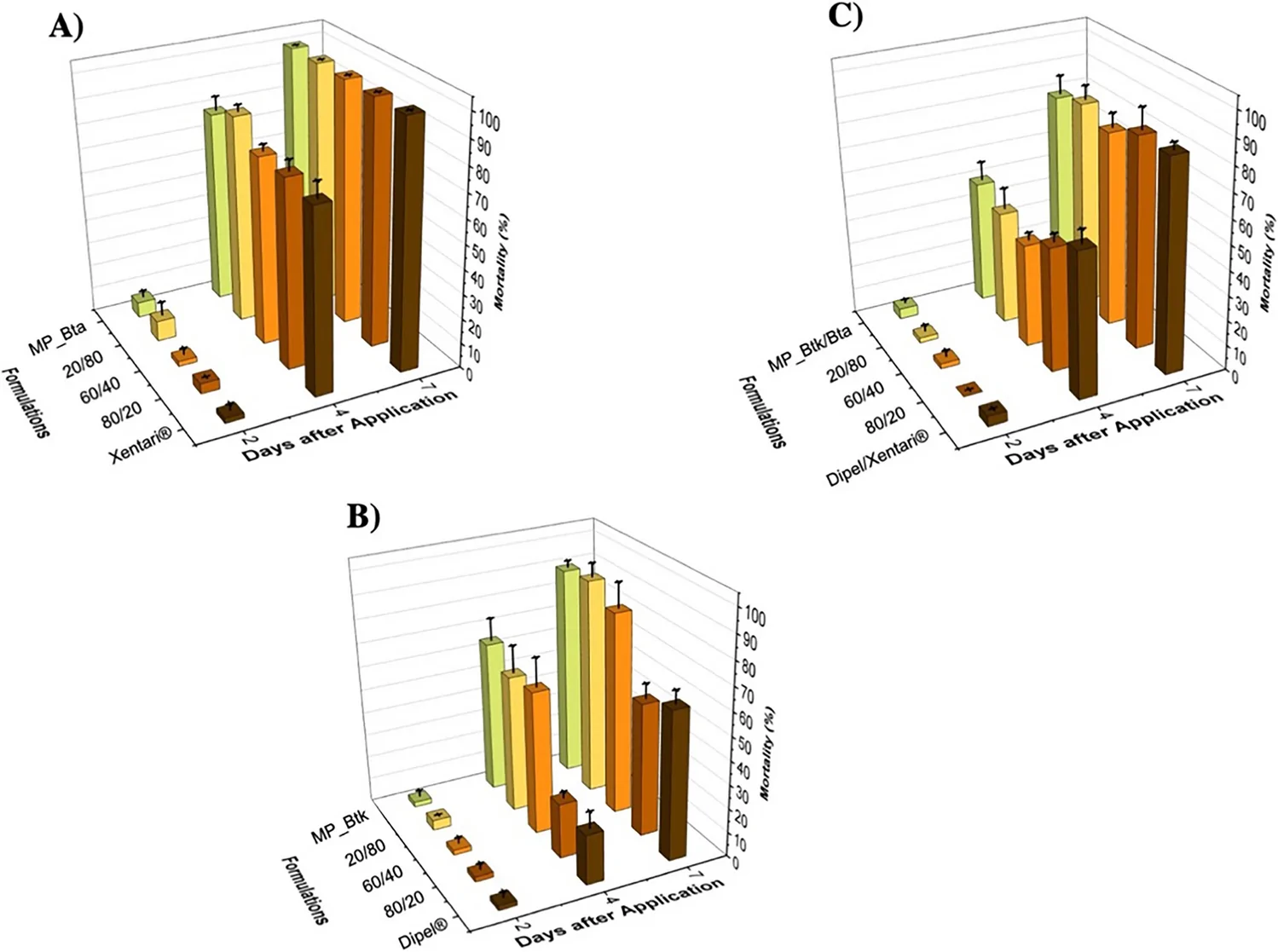
Effect of UV radiation on the toxicity of different Bt formulations against *S. frugiperda* larvae. **A** Xentari^®^ formulation and mixtures with different proportions of MP_Bta; **B** Dipel^®^ formulation and mixtures with different proportions of MP_Btk; **C** Dipel®/Xentari^®^ formulation and mixtures with different proportions of MP_Btk/Bta

The different formulations (14 µg formulation/cm^2^ diet) were exposed to UV radiation, and mortality tests were performed. Figure 4 shows the *S. frugiperda* mortality data evaluated after 2, 4, and 7 days. The results of the negative control (UV radiation to plates with only water applied to the surface of the diet) demonstrated that over the course of the analysis period, there was no negative effect on larval mortality, with a mean mortality rate of 4 ± 1.2%. These values are deemed suitable for toxicity assay controls.

Importantly, the Bta strain in the commercial product Xentari^®^ provides effective control of *S. frugiperda*. Our data showed that Xentari^®^ and its respective microencapsulated formulation MP-Bta had a similar insecticidal activity after UV irradiation. However, we can observe differences between the Dipel^®^ formulation and its respective microencapsulated formulation MP-Btk (Fig. 4B). A great difference was observed for these formulations after 4 days of application, and samples with a higher concentration of microencapsulated product had higher mortality up to 75% mortality than Dipel^®^, which induced 20.8% mortality. These values reached 87.5 and 66.7% after 7 days, respectively, indicating a better UV damage protection of MP_Btk microencapsulation, resulting in higher mortality of the larvae. Furthermore, the treatment containing the proportion of 60% of MP_Btk and 40% of commercial Dipel^®^showed mortality values of 4.2, 87.5, and 95.8% after 2, 4, and 7 days, respectively, consisting of the most effective combination of formulations.

## Discussion

In this study, we analyzed the different steps in the mechanism of action of Cry toxins to determine whether the formulation itself can affect the performance of Bt Cry toxins. Specifically, we compared commercial non-encapsulated products with microencapsulated products named MP_Bta and MP_Btk. The same Bt strains were used as the base material to make Xentari^®^ and MP_Bta or Dipel^®^ and MP_Btk. According to the manufacturer, Dipel^®^ is formulated with Btk and has the proteins Cry1Aa (15%), Cry1Ab (39%), Cry1Ac (23%), and Cry2Aa (22%), while Xentari^®^ is formulated with Bta and has Cry1Aa (21%), Cry1Ab (53%), Cry1Ca (20%), and Cry1Da (6%) (Valent 2022).

Results showed that non-encapsulated commercial products had a higher concentration of total protein than microencapsulated formulations after suspension in a similar water volume. Similar protein concentrations of these formulations were then subjected to solubilization analysis. We found no changes in the profile of solubilized protoxin bands in the SDS-PAGE analysis, indicating that the matrices used in the microencapsulation process did not influence the solubilization of Cry proteins. However, Table 1 shows that all tested formulations did not have 100% solubilization efficiency. Importantly, these formulations (conventional and microencapsulated) present a mixture of Cry toxins, which may influence their solubilization process. Aronson et al. (1991) tested different solubilization buffers for Bta and Btk strains and obtained solubilization efficiencies ranging from 8 to 70%. The authors concluded that the differences in solubilization between strains might be related to the protoxin composition of the crystals. Our data confirmed that the Btk strain showed higher solubilization efficiency compared to the Bta strain. Variations in solubilization values were observed regarding conventional and microencapsulated formulations, in which microencapsulated formulations had lower solubilization values under in vitro conditions. However, microencapsulated formulations showed better solubilization than conventional formulations when midgut juice was used to solubilize these samples.

The solubilization of Bt parasporal crystals is an essential step in the intoxication process, facilitated by the physicochemical conditions (mainly pH and reducing conditions) found in the host’s digestive fluids (Gill et al. 1992; Bravo and Soberón 2008; Deist et al. 2014). Du et al. (1994) compared two strains (Bta and Btk) for the solubility of their parasporal crystals. The authors showed that the solubilization started with pH values of 9.5 for both strains, reaching complete solubilization only when the pH reached values above pH 11 and proposed that similar high pH conditions should be found inside the midgut lumen of the lepidopteran larvae. The authors also described that the insecticidal crystals presented distorted and destabilized disulfide bonds, which can also influence the processes of solubilization and toxicity. Naimov et al. (2008) (Naimov et al. 2008) evaluated the solubilization of Bt *thompsoni* HD542 crystals composed of Cry15Aa protein. The authors tested different buffers and pH values (ranging from 6.0 to 11.0) with or without the reducing agent DTT and obtained complete protein solubilization only at pH 11.0 in carbonate and in CAPS buffers. The reduction of disulfide bonds allowed solubilization at pH 10. Other buffers (ethanolamine, Tris, and borate-buffered saline) did not solubilize a significant amount of protein with or without DTT addition. Our data indicated that the midgut lumen in *S. frugiperda* had a pH of 9.5, and that solubilization is low under this condition. The complete solubilization and activation of Cry proteins could only be observed after increasing the pH up to 10.5. However, our results also demonstrate that incubation time is determinant for solubilization. We reinforce the importance of studying additional factors, which may contribute to altering the midgut pH values, which could impact the solubility and toxicity of these proteins, such as the different instars and the geographic region where the pest can be found (Bravo and Soberón 2008). It may be worthwhile to isolate in the future some Cry mutants with improved solubilization, especially at lower pH values, which may correlate with improved toxicity.

During the activation step, approximately 40–60 amino acids of the N-terminus of 70 kDa and 130 kDa protoxins are removed by proteases. In addition, the 130 kDa protoxins showed to be cleaved out at the C-terminus (about 500–600 amino acids), and in both cases, the activated Cry toxins resulted in ~ 55 to 65 kDa (Bergamasco et al. 2013; Bravo et al. 2002; Gómez et al. 2014). Here, the activated proteins in Bta and Btk were correctly activated, and no great difference was observed in the proteolytic patterns of the different formulations. A double band of ~ 70 and ~ 65 kDa was observed for formulations containing a mixture of Cry1, and a band of ~ 55 kDa was verified for formulations containing Cry2. Liu et al. (2020) (Liu et al. 2020) obtained similar proteolytic profiles when evaluating the activation of Cry1Ac and Cry2Ab protoxins by proteases from the midgut juice of *Helicoverpa armigera* larvae. Our data showed that the profile observed for the cleavage with chymotrypsin differs from the other treatments, being possible to observe with more precision the ~ 55 kDa bands in formulations containing Cry2 protoxin. However, the protein profiles obtained after incubation with trypsin and midgut juice were very similar, supporting that midgut juice contains high trypsin levels and low chymotrypsin levels. In this sense, Saadaoui et al. (2009) (Saadaoui et al. 2009) showed that trypsin-like activity is predominant in the midgut homogenate of *Ephestia kuehniella*. However, the activation step has been related to resistance mechanisms. Inadequate activation, such as insufficient processing or excessive digestion, can result in insect resistance to the action of the Cry protoxin (Domínguez-Arrizabalaga et al. 2020).

Several studies in the literature have reported that Cry toxin receptors are located in BBM, including cadherin-like (Aimanova et al. 2006; Zhang et al. 2020; Jin et al. 2021), aminopeptidase N (APN) (Wei et al. 2016; Shao et al. 2018), and alkaline phosphatase (ALP) (Likitvivatanavong et al. 2011; Stalinski et al. 2016). The activated toxin samples from the different formulated products analyzed in this work showed similar binding to BBMV from two lepidopteran insects. Bel et al. (2017) (Bel et al. 2017) studied the binding of different Cry toxins (Cry1Ab, Cry1Ac, Cry1B, Cry1C, and Cry2Ab) with BBMVs from different insects (*S. exigua*, *S. litura*, *A. ipsilon*, and *H. armigera*) and reported different binding curves for these toxins, showing that these toxins exhibit different affinity values for BBMVs from different larval species. Our studies analyzed a mixture of activated toxins, and we could not provide specific affinity values for each protein.

Bioassay data against *M. sexta* and *S. frugiperda* larvae supported that the different formulation did not affect the mechanism of action of Cry toxins, as no differences in LC_50_ values were observed between commercial and microencapsulated products. These tests were carried out under laboratory conditions as reported by Eski et al. (2019), where microencapsulated formulations of an indigenous Bt Se13 strain were evaluated against *S. exigua*, showing similar LC_50_ values compared to commercial formulations.

However, our data indicate that the toxicity of the Dipel® formulation treated with UV and analyzed against *S. frugiperda* larvae was lower than the MP_Btk formulation. Microencapsulation helped to maintain the insecticidal effect of Btk, probably due to improved protection to UV damage of crystals and spores. Moreover, the results showed that the combination of conventional and microencapsulated formulations can be an important management strategy.

Khorramvatan et al. (2014) (Khorramvatan et al. 2014) compared the effect of three polymers (starch, gelatin, and sodium alginate) for the production of a microencapsulated Bt formulation. The authors observed that the spore viability was 90% after exposure to long-term UV radiation (UVB 385 nm) for the alginate polymer, while the viability of non-microencapsulated spores under this condition was only 40%. In another study, Jalali et al. (2020) used the Pickering-emulsion technique to perform the Bt microencapsulation. The authors tested different materials such as latex particles, graphene oxide nanosheets, and olive oil as protective materials and evaluated these formulations against *E. kuehniella* larvae after UV-A radiation. Their results showed that the combination of matrices at a concentration of 0.045% allowed an effective control and higher protection against UV radiation, corroborating the effectiveness of microencapsulation.

The results presented in this study demonstrate that the solubilization of conventional and microencapsulated formulations after ingestion is a critical point, and it seems to be relatively inefficient in vivo. Microencapsulated products showed a slightly higher solubilization efficiency. However, these differences were not significant when analyzing toxicity, as both types of formulations presented similar toxicity values. The conclusion is that the initial steps in the mechanisms of action of these pesticidal proteins (solubilization, activation, and binding to receptors present in BBMVs) were not significantly affected due to the microencapsulation procedure.

In addition, our data showed that microencapsulated formulations subjected to external factors, such as UV radiation, had greater control efficiency in shorter times. We propose that the mixture of conventional and microencapsulated formulations may be an important management strategy for the development of future biopesticide formulations. It is important to mention that UV light irradiation in environmental conditions may last longer and may involve a combination of UV-A and UV-B radiation. Our aim in this study was to establish a proof-of-concept for the technology and evaluate the protective capacity of microencapsulation. The data presented in this work support that Bt microencapsulated formulations provide UV protection showing to be a valuable eco-friendly approach to *S. frugiperda* control in regions with height temperatures and dry seasons, such as Africa and Asia, where this insect was recently reported and considered a threat for the food security (Mendesil et al. 2023; Tay et al. 2023).

## Supplementary Information

Below is the link to the electronic supplementary material.

Supplementary file1 (PDF 720 KB)

## Data Availability

Data are available from the authors upon reasonable request before release data.
