# Supplementary material for: Performance insights into spray-dryer microencapsulated Bacillus thuringiensis cry pesticidal proteins with gum arabic and maltodextrin for effective pest control
Source: Appl Microbiol Biotechnol. 2024 Jan 29;108:181. doi: 10.1007/s00253-023-12990-7 (PMC10824793; doi:10.1007/s00253-023-12990-7)

SUPPEMENTARY MATERIAL

Performance Insights into Spray-Dryer Microencapsulated *Bacillus thuringiensis* Cry Pesticidal Proteins with Gum Arabic and Maltodextrin for Effective Pest Control

Jhones Luiz de Oliveira[a,b], Isabel Gómez[b], Jorge Sánchez[b], Mario Soberón[b], Ricardo Antonio Polanczyk[a*] and Alejandra Bravo[b*]

[a] Department of Agricultural Production Sciences, Faculty of Agronomy and Veterinary Sciences, São Paulo State University (UNESP), Jaboticabal, São Paulo 14884-900, Brazil

[b] Department of Molecular Microbiology, Instituto de Biotecnología, Universidad Nacional Autónoma de Mexico, Cuernavaca, Morelos, Mexico

Figure S1: Evaluation of the protein profile in SDS-PAGE analysis. A) Protein profile of Bt *aizawai* (Bta strain) and Bt *kurstaki* (Btk and HD-1 strains) in different culture media (HCT, SP and Embrapa-EMB); B) Protein profile for non-encapsulated Bt formulations (1, Xentari®; 2, Dipel®; and 3, Dipel®/Xentari®) and microencapsulated samples (4, MP_Bta; 5, MP_Btk; and 6, MP_Btk/Bta).

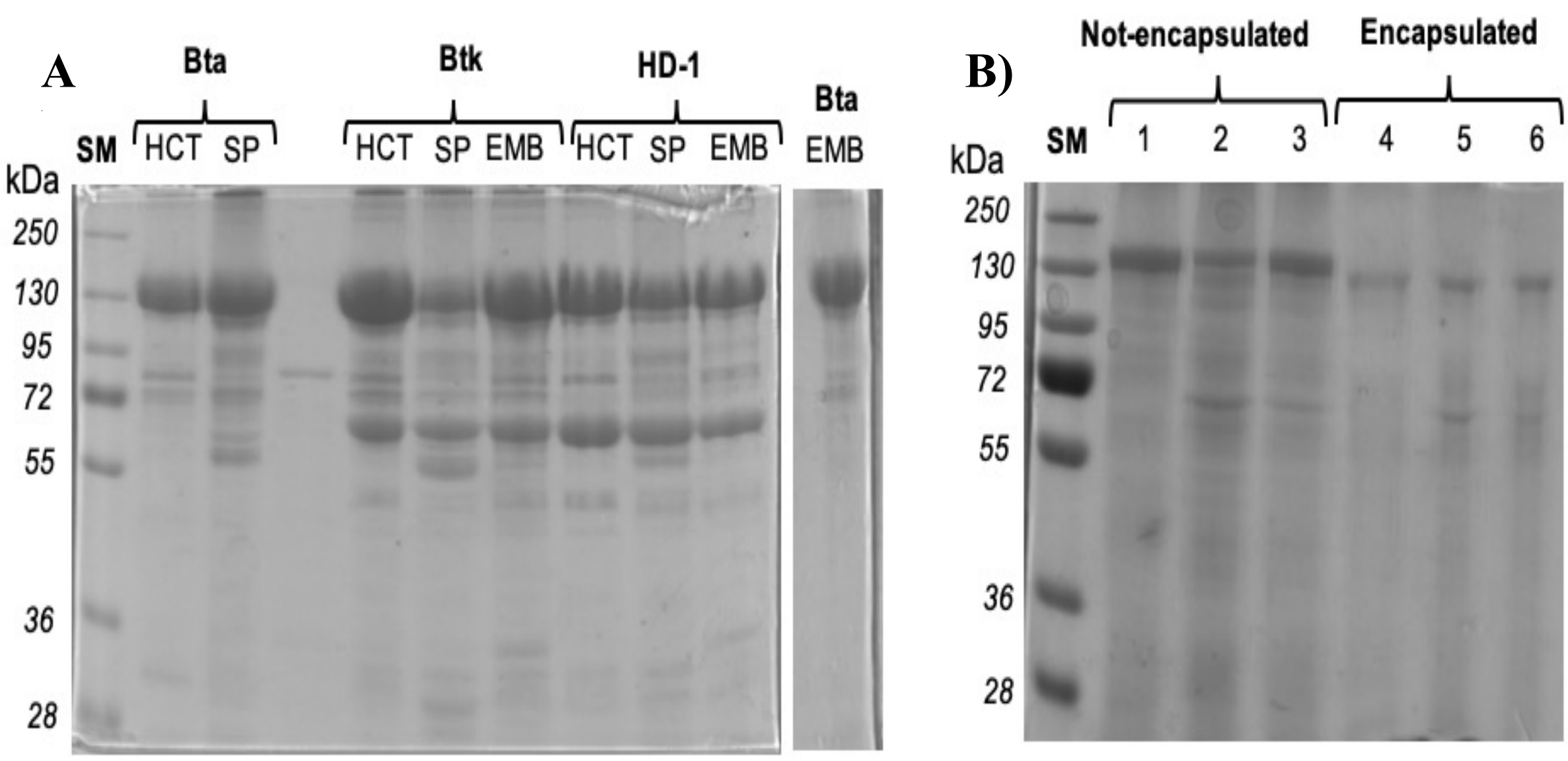

Figure S2: Analysis of activation efficiency after Protein quantification of SDS-PAGE profiles. A) Protein profile after activation with trypsin; B) Protein profile after activation with chymotrypsin; C) Protein profile after activation with midgut juice; Formulations: 1, Xentari®; 2, Dipel®; 3, Dipel®/Xentari®; 4, MP_Bta; 5, MP_Btk; and 6, MP_Btk/Bta.

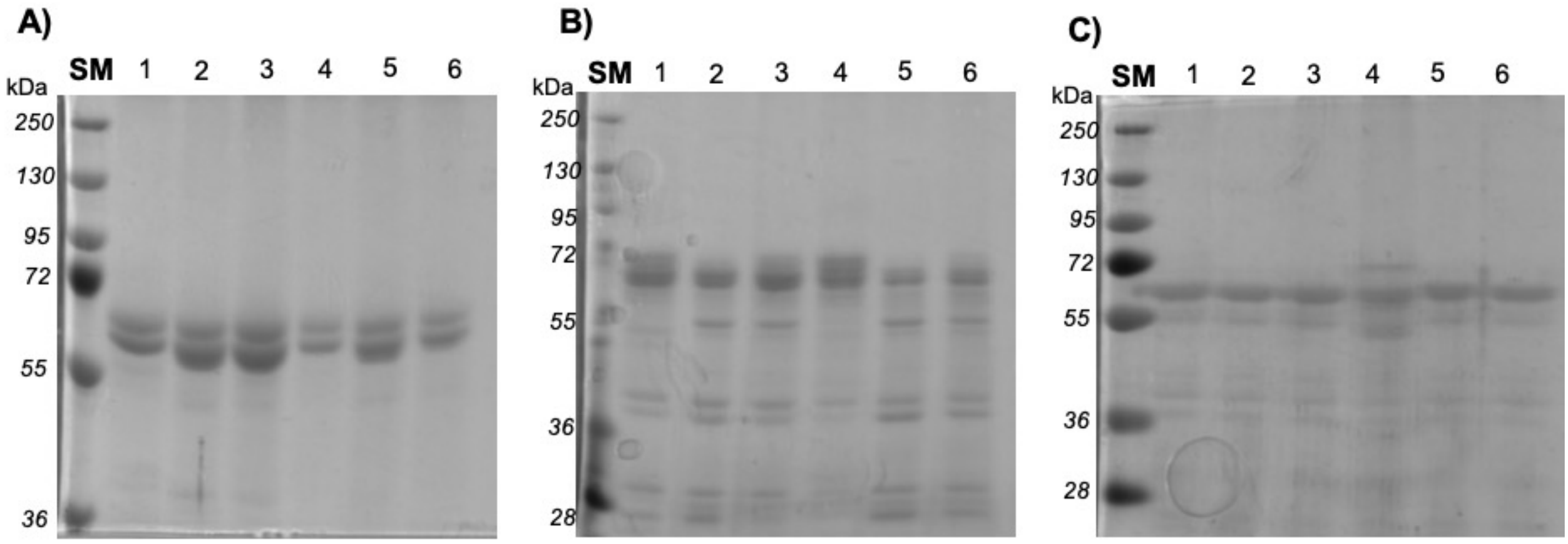

Figure S3. Binding interaction of formulations to BBMV from *Spodoptera frugiperda* (A and B) and *Manduca sexta* (C and D). A) A sample of 10 µg SfBBMV was incubated with 1 to 15 µg of Cry activated toxins present in Xentari and MP_Bta formulations. B) A sample of 10 µg of SfBBMV was incubated with 1 to 15 µg of activated toxins present in Dipel and MP_Btk formulations. C) A sample of 10 µg of MsBBMV was incubated with 1 to 15 µg of toxins present in Xentari and MP_Bta formulations. D) A sample of 10 µg of MsBBMV was incubated with 1 to 15 µg of activated toxins present in Dipel and MP_Btk formulations.

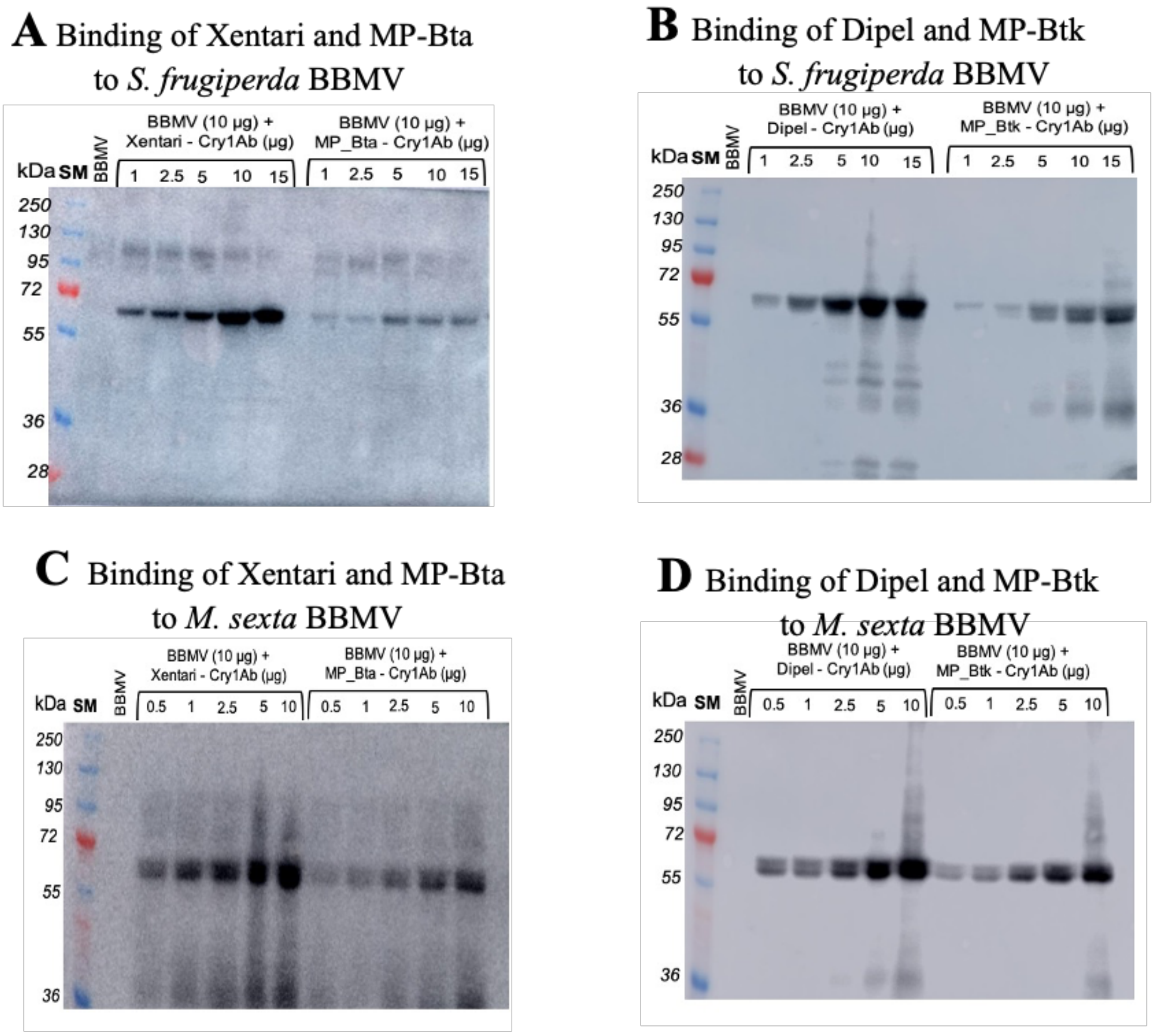

Figure S4: ELISA binding analysis of Xentari, Dipel, MP_Bta and MP_Btk formulation containing trypsin-activated Cry1Ab toxin to SfBBMV (A) and MsBBMV (B).

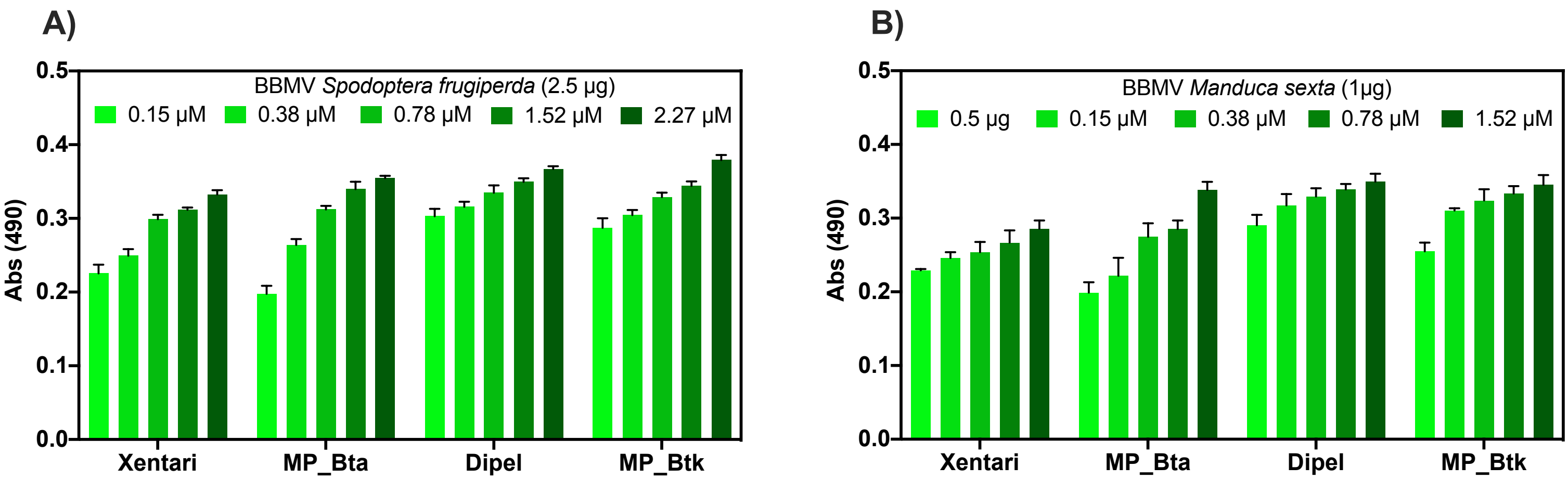

Supplement: Supplementary file 1 [file 253_2023_12990_MOESM1_ESM.pdf]
